# Dermatofibroma Arising within a Black Tattoo

**DOI:** 10.1155/2014/745304

**Published:** 2014-09-23

**Authors:** Alejandro Lobato-Berezo, Micaela Churruca-Grijelmo, Marcela Martínez-Pérez, Adrián Imbernón-Moya, María Elena Vargas-Laguna, Eva Fernández-Cogolludo, Antonio Aguilar-Martínez, Miguel Ángel Gallego-Valdés

**Affiliations:** Department of Dermatology, Hospital Universitario Severo Ochoa, Avenida Orellana s/n, Leganés, 28911 Madrid, Spain

## Abstract

Many complications have been reported over tattoos, some of which are tumours, such as dermatofibromas. It is important to establish a differential diagnosis because they can resemble other malignant lesions as dermatofibrosarcoma protuberans. We report the development of a dermatofibroma in a 21-year-old man with a tattoo painted two years ago.

## 1. Introduction

Tattooing consists in the introduction in the dermis of indelible and exogenous pigments by needle and may be associated with complications such as infections, inflammatory and granulomatous reactions, as well as neoplasms [1].

## 2. Case Report

**Figure 1:**
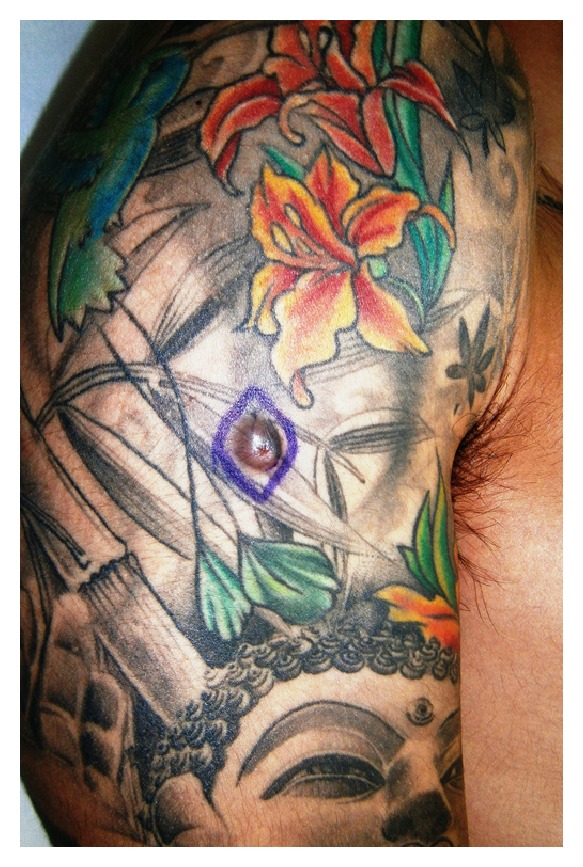
Dermatofibroma: 10 mm nodule arising in a black tattoo.

**Figure 2:**
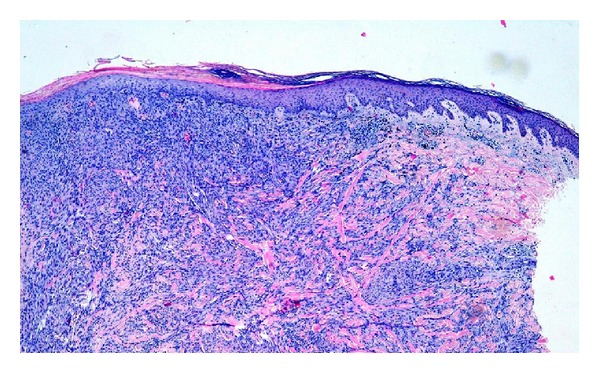
Dermal proliferation of fibroblastic-like cells and black pigment.

A 21-year-old man was referred to the emergency department with a 2-month history of a painful lesion arising from a black tattoo he had been done 2 years ago. He had no significant past medical history. The physical examination revealed a nodular lesion measuring 10 mm involved in a black tattoo on his right arm. The nodule was firm, tender, mobile, and light-red coloured (Figure 1). A skin biopsy was performed and showed a nodular proliferation in the dermis consisting of very numerous collagen and fibroblastic-like cells in an irregular arrangement. No cytological atypia was found. Extracellular deposits of black ink pigments were observed within the proliferation. The epidermis was acanthotic with basal hyperpigmentation (Figure 2). Immunohistochemistry was positive for factor XIIIa and CD-68 and negative for CD34, S-100, and actin. Surgical removal was performed with no evidence of recurrences.

## 3. Discussion

Benign and malignant tumors, such as seborrheic keratosis, keratoacanthomas [2], melanomas, epidermal cysts, squamous cell carcinomas, basal cell carcinomas, and dermatofibrosarcoma protuberans [3] are well-recognized conditions but uncommonly arising over decorative tattooing. Dermatofibroma is a common benign soft tissue tumor of the skin with a controversial origin. The condition is considered to be a reactive or postinflammatory process and may occur after local trauma in 20% of cases. In fact, there are reports of dermatofibromas arising after blunt, nipple-piercing, insect bite, and vaccination scars. Dermatofibromas arising over tattoos have been reported in three patients and the lesions developed several months after the placement in the tattoo, located in the arms or in the legs [4, 5].

The association between dermatofibromas and tattoos may be coincidental, given the great number of people with tattoos nowadays and the anecdotal presence of these tumors. However, these two conditions have a link because the skin was free of any type of lesion before the tattoo and there is a clear chronology between the development of the dermatofibroma and the tattoo. Furthermore, dermatofibromas can appear after trauma, and tattooing represents an action which triggers a nonspecific inflammatory reaction at punctured areas in an attempt to eliminate the toxic or carcinogenic agents contained in the ink. This process may induce a local fibrosis reaction.

Dermatofibroma is a frequent tumor and the number occurring in tattoos is very rare. Evidence for a clear causal relationship is lacking due to limitations imposed by the relative rarity of these tumors arising within tattoos and by the nature of retrospective analysis. Therefore, the extent of this association and its underlying mechanisms remains uncertain. However, we suggest that dermatofibroma should be included in the differential diagnosis of tumors arising in sites of tattoos.
